# Attentional asymmetries – cause or consequence of human right handedness?

**DOI:** 10.3389/fpsyg.2014.01587

**Published:** 2015-01-13

**Authors:** Gavin Buckingham, David P. Carey

**Affiliations:** ^1^Department of Psychology, School of Life Sciences, Heriot-Watt UniversityEdinburgh, UK; ^2^School of Psychology, Perception, Action and Memory Research Group, Bangor UniversityBangor, UK

**Keywords:** handedness, laterality development, bimanual coordination, attention, motor control, laterality of motor control

## Abstract

It is well established that the vast majority of the population favors their right hand when performing complex manual tasks. However, the developmental and evolutionary underpinnings of human manual asymmetries remain contentious. One often overlooked suggestion is that right handedness may stem from an asymmetrical bias in attention, with the right hand being allocated more attentional resources during bimanual tasks than the left hand (Peters, 1981). This review examines the evidence for attentional asymmetries during a variety of bimanual tasks, and critically evaluates the explanatory power of this hypothesis for explaining the depth and breadth of individual- and population-level manual asymmetries. We conclude that, while the attentional bias hypothesis is well-supported in adults, it requires further validation from a developmental perspective to explain the full breadth of adult manual laterality.

Approximately 90% of humans consider themselves to be right handed (Coren and Porac, 1977). This unique manual asymmetry can be taken to have at least two related, but not entirely overlapping, meanings: (1) a higher level of skill when using the right hand for complex manual tasks and (2) a preference to select the right hand to perform most daily activities. Competing explanations for the cause and consequences of human handedness have tended to emphasize the asymmetries of performance or selection, implying that one drives the other. Other explanations have suggested that handedness is a consequence of the leftward lateralization of language present in the majority of the population (Annett, 2000).

The goal of this article is to provide an overview of the evidence for a rarely discussed hypothesis – that right handedness is a consequence of a rightward attentional bias. Proponents of this hypothesis claim that when attention must be divided between the hands during bimanual tasks, most individuals will allocate the majority of their attentional resources to the right hand or its task. First, we describe the evidence for an automatic link between the attentional system and manual actions. Next, we present the empirical studies which have found evidence for a rightward attentional bias in right-handed individuals’ during rhythmic and discrete bimanual tasks. Finally, we discuss the viability of the attentional bias explanation as a way to bridge the gap between performance and selection asymmetries.

## ATTENTIONAL YOKING WITH HAND MOVEMENTS

On the face of it, there would seem to be obvious advantages to having two equally skilled hands to complete twice as many tasks. Such a strategy, however, would not be easily compatible with humans’ attentional limitations: prior to commencing a typical reach toward a visual target, a saccade is used to aim the high-resolution foveal portion of the eye to the region of interest (Desmurget et al., 1998; Flanagan and Johansson, 2003). Although this serial chain of events may seem obvious in a visually guided task, some evidence suggests that there is an automatic link between overt attention and action (for a recent critical review, see Smith and Schenk, 2012). Fisk and Goodale (1985) demonstrated that that saccades and hand movements toward visual targets are yoked together, with an eye movement’s onset driven by the latency of the hand movement’s onset. Similar conclusions have been drawn by Neggers and Bekkering (2000), whose experiment appeared to demonstrate that a new saccade cannot be planned until the preceding reach to a visually defined target has been completed. Furthermore, there is good evidence for a yoking between temporal aspects of hand and eye kinematics, with the time at which a saccade lands being well correlated with the time at which the reaching arm is at the point of peak acceleration (Helsen et al., 1998). Clinical evidence for a yoking between the eyes and hands comes from cases of ‘magnetic misreaching’ (Carey et al., 1997; Jackson et al., 2005), where patients with bilateral parietal lobe damage are unable to reach to any direction other than the target of their gaze (see also van Donkelaar and Adams, 2005). By contrast, Buxbaum and Coslett (1998) report an ataxic patient showing the opposite clinical sign, with the patient’s gaze becoming spontaneously fixed upon his hand during movements, interfering with goal-directed activities.

The link between attention and action in the context of physiology and neuropsychology is well-studied in the context of sequential unimanual movements (for review, see Baldauf and Deubel, 2010). There is, however, far less research examining how the attentional systems behave when both hands are moving simultaneously or being coordinated to complete a task.

## ATTENTIONAL ASYMMETRIES DURING RHYTHMIC BIMANUAL TASKS

The seminal study on attentional biases during bimanual coordination was undertaken by Peters (1981), who had participants tap one hand to the beat of a metronome (the easier task, with no inherent asymmetries) while the other hand tapped at its maximum rate (a more difficult task, where the dominant hand tends to excel) in a sample of left and right handers. Right-handed subjects experienced no difficulties when their right hand was performing the difficult rapid tapping task and their left hand was performing the easy metronomic tapping task. When tapping in the converse arrangement, however, the right handers suffered large performance decrements in both tasks. The critical link to attention can be inferred from the fact that it is not just the non-dominant hand which suffers, but rather that when the configuration is ‘wrong,’ both hands are equally impaired in their performance of their respective tasks. In other words, the poor performance seen in both the easy and difficult tasks when the left hand was assigned the more difficult job was a consequence of a rightward bias in attention rather than a motoric asymmetry.

This early demonstration of an attentional asymmetry has been followed by work examining subtle differences in between-hand coordination when participants are asked to move their hands back and forth synchronously at various frequencies. Treffner and Turvey (1995) examined right handers’ ability to move a large pendulum held in each hand forward and backward in simple coordinative patterns. The authors found a tendency for the right hand to slightly lead the left hand when participants were instructed to move their hands synchronously. The attentional nature of this asymmetry was clarified in later work by Amazeen et al. (1997), who showed that the right hand lead was reduced when attention was directed away from the right hand and the overall variability (i.e., SD of relative phase) of the rhythmic movements was increased when attention was directed away from the dominant hand. In other words, when right-handed subjects perform an inherently low variability task they instead tend to perform with a slight right hand lead which appears to reflect their prior bias in attention, which can then be manipulated by altering the direction of overt attention, at the expense of overall performance variability. Interestingly, attending toward the right hand during similar bimanual tasks has been shown to increase this phase lead and improve performance in terms of variance as compared to free viewing or attending toward the left hand (Swinnen et al., 1996; Rogers et al., 1998). Thus, the performance in these tasks can be modulated by shifting attention, with individuals performing best when attending their right hand and performing worst when attending to their left hand (see also Treffner and Turvey, 1996).

## ATTENTIONAL ASYMMETRIES DURING DISCRETE BIMANUAL TASKS

The most straightforward method of investigating attention during discrete bimanual movement has been to examine eye movements during bimanual reaches toward visual targets. Early work examining the horizontal direction of right-handed participant’s eye movements using electrooculography during rapid bimanual reaches noted that participants tended to direct their gaze toward the right side of space, either in isolation or prior to making a leftward saccade (Honda, 1982). More recently, Riek et al. (2003) examined the direction of gaze during bimanual reaches to target pairs. They noted that participants tended to make two eye movements during symmetrical reaches: one from fixation and a terminal saccade toward the leftward target, indicating that the right side of space was monitored for the duration of the reach (see also Srinivasan and Martin, 2010).

Given that overt and covert attention can be readily dissociated (Posner, 1980; Hunt and Kingstone, 2003), it is quite possible that the direction of attention could be preferentially biased one way or the other without movements of the eyes. To this end, Baldauf and Deubel (2008) examined how a small number of right handers performed a simple perceptual task at the goal locations of a bimanual reach. They noted that, although perceptual performance was enhanced at the target locations for both hands, there were no differences in discrimination ability between the targets of the right versus the left hand. This lack of asymmetry may be due to a lack of power, but may also suggest that any attentional asymmetry might manifest itself in motor, rather than perceptual outcomes.

To examine motoric aspects of an attentional asymmetry, we have undertaken several experiments to using a discontinuous double-step bimanual reaching task (Buckingham and Carey, 2009). This task was adapted from classic double-step paradigms (Goodale et al., 1986), and consisted of two discrete steps: a bimanual reach toward a pair of visual targets followed by a unimanual reach to a new target which appeared halfway through the bimanual reach in 25% of the trials. Participants had to complete the bimanual reach before they made a unimanual reach with whichever hand was closest to the newly appearing single target. An asymmetrical allocation of attention during the bimanual reach should have behavioral consequences for the downtime between the bimanual and unimanual portions of the task (i.e., the refractory period). We predicted that participants would be able to prepare and commence the reaches with the attended hand more rapidly than with the non-attended hand, which would presumably require a time-consuming attentional shift in its direction prior to commencing the reach. In a sample of right-handed individuals we noted a clear advantage for the right hand, which was able to initiate the unimanual portion of the task some 20 ms faster than the left hand. This right hand advantage is particularly interesting because it contrasts the normal pattern of asymmetries observed during unimanual reaching tasks, where the left hand typically reacts faster than the right hand (Boulinguez et al., 2001). In other words, our data suggest that a right hand unimanual localization reaction time advantage only exists when preceded by a bimanual movement. Not only was this asymmetry reversed when participants were told to explicitly focus their attention toward their left hand during the task, but this attentional manipulation reduced the right hand’s performance rather than improved the left hand’s performance. These findings suggest (somewhat counterintuitively) that attending one’s non-dominant hand may be a risky strategy for successful coordination of the hands.

To investigate how attentional biases may influence the propensity to select one hand over the other, we modified the double-step reaching task to include a hand selection cue (Bestelmeyer and Carey, 2004; Buckingham et al., 2011). As above, participants made a bimanual reach toward a pair of visual targets. Prior to this reach, however, they received a small vibratory cue to one of their hands to indicate which hand would have to perform the follow-up unimanual reach with 80% accuracy. The critical trials were when the cue was invalid (i.e., when the right hand was cued, but a left hand reach was required). Here, right-handed participants made more errors and a spent more time inhibiting the right hand when a left hand movement was required than the converse, suggesting that their right hand is pre-selected to undertake reaches. Left handers, by contrast, showed no such asymmetry, suggesting that they may lack any selection/attention bias whatsoever.

## THE LINK BETWEEN ATTENTIONAL ASYMMETRIES AND MANUAL LATERALITY

The studies outlined above have indicated that subtle asymmetries which can be easily ascribed to attentional affects seem to favor (or be directed toward) the right hand of right handers. However, these findings offer little insight into the causal relationship between attentional and manual asymmetries. Clearly, altering one’s hand preference is not simply a case overtly attending toward the non-dominant hand (Swinnen et al., 1996; Treffner and Turvey, 1996; Amazeen et al., 1997; Buckingham and Carey, 2009). It is, of course, also possible that attentional biases are a consequence, rather than the cause, of hand preferences. It is by examining attention and the emergence of manual laterality in a developmental context where the attentional bias hypothesis may succeed in breaking the cause and effect circularity which plagues theories of handedness.

The attentional bias hypothesis posits that attention is biased toward the right hand in a substantial proportion of the population from birth (Peters, 1981, 1991, 1994). This initial bias in attention may stem from the rightward orienting asymmetry which has been shown in human infants (Hopkins et al., 1987), and could lead to asymmetries in the roles assigned to either hand over the course of development. Continued use of the right hand as the performer of the more skilled portion of a dyadic task would then lead to inevitable right hand performance advantages as a function of practice. Little direct evidence for this causal link between attentional asymmetries and manual laterality exists, although there an increasing body of work indicating that attention can modulate the cortical underpinnings of motor learning, such as the generation of motor memories in primary motor cortex (Stefan et al., 2004). Furthermore, it is worth noting that infants are orientating their attention long before they are making purposeful movements, and some it has been established that the degree of rightward orienting bias seen in infancy does show a link to the development of manual asymmetries across childhood (Michel and Harkins, 1986). Indeed, a recent study has demonstrated that occluding the preferred arm of infants who have recently started reaching toward objects, results in a shift of their manual preference away from the occluded hand (Pogetti et al., 2014). However, longitudinal evidence for a link between attentionally modulated behavioral asymmetries during bimanual tasks in childhood and later-life unimanual hand preference would seem necessary to confirm the causal relationship between these factors.

While the link between consistent right hand selection and right hand performance advantages is easy to understand, it is worth considering why an attentional asymmetry is necessary in human motor coordination. Peters suggests that the key to the attentional bias hypothesis is bimanual coordination – the common factor linking the experiments described in this review. The ‘kinematic chain’ hypothesis, proposed by Guiard (1987) builds on the supposition that the majority of goal-directed actions are, to a degree, bimanual. In adults this bimanual coordination is often be symbolic or supportive in nature, with one hand facilitating the other’s behavior (e.g., the left hand framing the face, while the right hand shaves with the razor blade). However, bimanual coordination is particularly prevalent in infancy where a combination of factors, such as a lack of motor skill and failure to inhibit mirror movements, ensures that bimanual interaction is the norm rather than the exception (for review, see Haywood et al., 2012). Developing from the simple reach-to-grasp behavior of infants to the complex goal-directed actions of adults, hand choice becomes a more complex matter of task assignment. One hand must be selected for a dominant role, whereas the other must be allocated a supporting role. It is this through the indirect link which an attentional asymmetry would drive adult handedness, linked by way of consistent selection biases which persist into unimanual variants of a multitude of tasks. Some tacit support for this proposition comes from Kourtis et al. (2014), who provided behavioral and electrophysiological evidence that performance in bimanual tasks with asymmetrical demands reflects the consistency, rather than the direction, of an individual’s handedness.

Another point which is worth consideration is how to reconcile the rightward attentional bias which right handers exhibit during bimanual tasks with the oft-reported left hand unimanual reaction time advantage during unimanual localization tasks (Boulinguez et al., 2001), which may be related to right hemispheric attentional mechanisms that facilitate disengaging from fixation, or moving attentional resources toward suddenly appearing visual targets (Mieschke et al., 2001). Indeed, the attentional bias toward the right hand discussed throughout this review might seem counterintuitive, given the evidence for right hemispheric lateralization for attention in the human brain (Petersen and Posner, 2012). Separable mechanisms for attention (related to stimuli in the external world) and “intention” (or motor attention, related to selecting relevant and inhibiting irrelevant actions; e.g., Main and Carey, 2014) might go some way toward reconciling these viewpoints. For example, Rushworth et al. (2001) have found evidence suggesting that this motoric attention is not only independent from visuo-spatial attention, but appears to be lateralized to the opposite cerebral hemisphere.

## ATTENTIONAL ALLOCATION IN LEFT HANDERS

Up until this point, only evidence has been presented from right-handed individuals. The situation for the understudied left handers remains unclear, largely because studies examining adextrals in this context are rare. In fact, they are crucial, if establishing the relationship to cerebral asymmetries is desired (see Carey and Johnstone, 2014, for review). In their study examining rhythmic coordination of the hands during synchronous pendulum swinging, Treffner and Turvey (1995, 1996) noted that left handers have the opposite pattern of asymmetries of right handers (i.e., a slight left hand phase lead, which is exacerbated at higher movement frequency). However, in Peters’ (1981) bimanual tapping task and the cued bimanual reaching task of Buckingham et al. (2011), there was no clear evidence of any asymmetries. This does not mean that the left handers tended to perform equally well with both hands, but rather that there tended to be equally sized sub-groups who performed better with one hand (or configuration) than the other, canceling one another out. In other words, left handers appear to lack population-level asymmetry seen in right handers. Typically, across a variety of behavioral metrics, left handers tend not to be the mirror image of right handers (Bryden, 1982; Carey and Johnstone, 2014). Instead, left handers are typically more ambidextrous and variable in their hand preferences, with only a small proportion showing the same degree to asymmetries as right handers. Given the relatively non-asymmetrical nature of this population, left handers may be a subset of individuals who lack a rightward attentional bias, forming a Gaussian distribution around which hand they select for a particular task. With no external biases to select one hand over the other (although this point clearly is a contentious one in what is often described as a right-handed world) would lead the average ‘unbiased’ individual to select their left hand for half of the tasks they typically perform, and their right hand for the other half.

## CONCLUSION

The underlying cause of human handedness is the cause of much debate. Here, we have presented the evidence for a bias in attention which occurs during bimanual coordination which may drive hand preferences, and presented a plausible account of how this bias would lead to manual asymmetries across the population. Future work on relating these effects to cerebral asymmetries in right and left handers, and how attentional and manual asymmetries develop and interact over developmental trajectories, may help clarify these relationships.

## Conflict of Interest Statement

The authors declare that the research was conducted in the absence of any commercial or financial relationships that could be construed as a potential conflict of interest.
